# Identification and Characterization of a New Autoimmune Protein in Membranous Nephropathy by Immunoscreening of a Renal cDNA Library

**DOI:** 10.1371/journal.pone.0048845

**Published:** 2012-11-08

**Authors:** Fabrizio Cavazzini, Riccardo Magistroni, Luciana Furci, Valentina Lupo, Giulia Ligabue, Maria Granito, Marco Leonelli, Alberto Albertazzi, Gianni Cappelli

**Affiliations:** 1 Department of Medicines, Emergency Medicine and Medical Specialities, Division of Nephrology Dialysis and Renal Transplantation, University of Modena and Reggio Emilia, Modena, Italy; 2 Division of Nephrology Dialysis and Renal Transplantation, Modena University Hospital, Modena, Italy; Institut Jacques Monod, France

## Abstract

Membranous Nephropathy (MN) represents a large amount of Nephrotic Syndromes in the adult population and its definitive diagnosis is currently carried out through biopsy. An autoimmune condition has been demonstrated in idiopathic MN (iMN) in which some kidney structures are targeted by patient autoantibodies. Some candidate antigens have been described and other likely involved target proteins responsible for the disease are not known yet. In this work our aim is to identify these proteins by screening a lambda-phage library with patients’ sera. We enrolled four groups of patients: two MN groups of 12 full iMN patients; one control group of 15 patients suffering from other renal diseases; one control group of 15 healthy individuals. A commercial cDNA phagemide library was screened using the above described sera, in order to detect positive signals due to antigen-antibody bond. We detected one phagemide clone expressing a protein which was shown to be targeted by the antibodies of the iMN sera only. Control sera were negative. The sequence analysis of cDNA matched the Synaptonemal Complex protein 65 (SC65) coding sequence. Further proteomic analyses were carried out to validate our results. We provide evidence of an involvement of SC65 protein as an autoimmune target in iMN. Considering the invasiveness and the resulting risk coming from renal biopsy, our ongoing aim is to set a procedure able to diagnose affected patients through a little- or non-invasive method such as blood sampling rather than biopsy.

## Introduction

Membranous Nephropathy (MN) is the most common cause of nephrotic syndrome in adults [1], [2]; it can be secondary to other clinical conditions including infections, autoimmune diseases, cancer and some toxic substances or drugs. However in most cases (about 80%), MN is classified as idiopathic (iMN), since the etiology of the nephropathy is still substantially unknown. Although spontaneous remission is observed in about one third of subjects, 40% of the patients develop end-stage renal failure after about 10 years. [2], [3] In histological preparations, a thickening of the glomerular basal membrane is observable as a consequence of subepithelial deposits of immune complexes with immunoglobulins predominantly belonging to the IgG_4_ subclass. [4], [5] Such complexes are apparent in immunofluorescence conducted with anti-IgG antibodies and in electron microscopy as subepithelial electron-dense deposits. A pivotal role in the events causing the glomerular lesions is played by the activation of complement and the assembly on the podocyte surface of the membrane attack complex C5b-9 that may be triggered by the same immunodeposits. [6], [7], [8].

The autoimmune nature of the disease is thus strongly suspected. This theory is supported by an abundant and consistent scientific literature that started in the late fifties with the description of the Heymann nephritis model. [9], [10], [11] The autoantigen target of this model was identified in the rat podocyte membrane protein named megalin. However, megalin has been found neither in human podocyte nor in human MN immunodeposits and therefore it could not be proved responsible for the human form of iMN. [12] The first antigen demonstrated to be clearly involved in the human disease is neutral endopeptidase (NEP), reported in some cases of antenatal membranous glomerulonephritis. [13], [14] This is a rare form of MN that can arise in newborns from mothers carrying a genetic deficiency of NEP: the protein is expressed by the podocytes of the embryo in the uterus, and NEP deficient mothers produce anti-NEP antibodies since their immune system recognizes it as a non-self protein. These data supported the theory that podocytes act as a source of antigens involved in the *in situ* formation of subepithelial immune complex deposits. More recently, these data were confirmed (by reports) by L.H. Beck *et al*. [15], M. Prunotto *et al.* [7] and M. Bruschi *et al* [16]. The first group demonstrated that sera from iMN patients show specific immunoreactivity against the M-type phospholipase A_2_ receptor (PLA_2_R) in about 70% of cases. This glycoprotein is expressed by podocytes and colocalizes with IgG in the subepithelial immune complex deposits. The second group (including the last two papers cited above ) found that aldose reductase (AR), manganese superoxide dismutase (SOD2) and α-enolase (ENO1) are targeted by iMN patients IgG_4_, thus effectively adding three more targets to the iMN antigen list.

On the basis of evidence of an autoimmune pathogenesis of the disease consisting in autoantibodies directed to primitive renal antigens and considering that NEP, AR, SOD2, ENO1 and PLA_2_R do not exclude a possible role for other antigens [17], we looked for patients sera reactivity to a wide panel of renal proteins. In particular, we screened a large number of lysis plaques from a lambda phage cDNA expression library comparing MN and control patients sera. Candidates were further validated by immune screenings and immunofluorescence techniques.

**Table 1:** Clinical characteristics of patients.

Parameter	Patient																							
	1	2	3	4	5	6	7	8	9	10	11	12	13	14	15	16	17	18	19	20	21	22	23	24
**Gender**	M	M	M	M	F	F	M	F	M	F	M	M	M	M	M	F	M	F	F	F	F	F	F	M
**Age at diagnosis**	44,4	77,3	37,7	75,9	63,6	68,5	40,7	49,1	66,4	72,6	88,7	75,1	21,4	69,3	77,1	48	64	70	29,2	85	76	67,6	38,6	50,6
**Histologic stage**	I-II	I	II-III	I-II	II-III	II-III	III	I	I	II-III	II-III	I	III	I	I-II	I	II	III	I	I	II-III	I	II-III	II
**Serum creatinine (mg/dl)**																								
** at diagnosis**	1,08	1,32	1,33	1,43	0,83	1,23	1,14	0,95	0,88	0,86	1,55	1,5	1,1	2	2	0,6	1,08	1,09	0,52	1	1,7	0,65	0,79	0,88
** after 1 year**				1,69	0,87		0,85	1,09	0,92		1,45	1,2	0,86	2,06	1,13	0,66	1,74							
**Serum albumin**																								
** at diagnosis**	2,5	2,42	2,8	2,45	2,74	2,2	2	2,46	2,58	2,83	2,74	2,6	2,81	2,21	1,7	2,5	3,7	2,88	3,35	2,3	2,2	2,9	3,29	3,05
** after 1 year**				4,3	4,53		3,99	2,77	2,53		3,99	3,71	3,58	1,97	4,01	4,42	3,15							
**Proteinuria**																								
** at diagnosis**	3,5	3	4,8	4,5	3,3	6,61	8	2,28	7,65	8	4,83	6,69	2,55	4,4	4,4	10	5,9	1,2	2,3	11,5	3,9	4,4	4,9	3,5
** after 1 year**				0,69	0,93		1,38	1,62	0,95		1	2,7	1,9	25	0,1	0	5,3							
**Anti-HCV**	NA	N	N	N	N	N	N	N	NA	N	NA	N	N	N	N	N	N	N	N	N	N	N	N	N
**Immunosuppressant therapy**	Y	Y	Y	Y	Y	Y	Y	Y	Y	Y	Y	Y	Y	Y	Y	Y	Y	Y		Y	Y	Y	Y	Y
**ACEI/ARB**	N	Y	Y	Y	Y	Y	Y	Y	Y	Y	Y	Y	N	N	Y	N	N	Y	NA	Y	Y	Y	Y	Y

## Methods

### Patients and Control Subjects

Twelve nephrotic patients affected by iMN for the discovery phase and twelve nephrotic patients affected by iMN for the confirmation phase were recruited. The diagnosis of iMN was made on the basis of biopsy examination using immunofluorescence techniques and optical microscopy according to the parameters of the ‘*World Health Organization Centre for the Histological Classification of Renal Diseases, 1982*’. Patients with the following characteristics were included in this study: biopsy-proven iMN, age older than 18 years, proteinuria >2 g/day, creatinine <1.8 mg/dL. Patients affected by connective-tissue diseases, positive to ANA, ENA antiDNA, antiphospholipids antibodies, ANCA, diabetes mellitus, neoplasia, Hepatitis B or C virus infection, HIV or systemic diseases, patients younger than 18 years at the time of biopsy were excluded.

**Figure 1:**
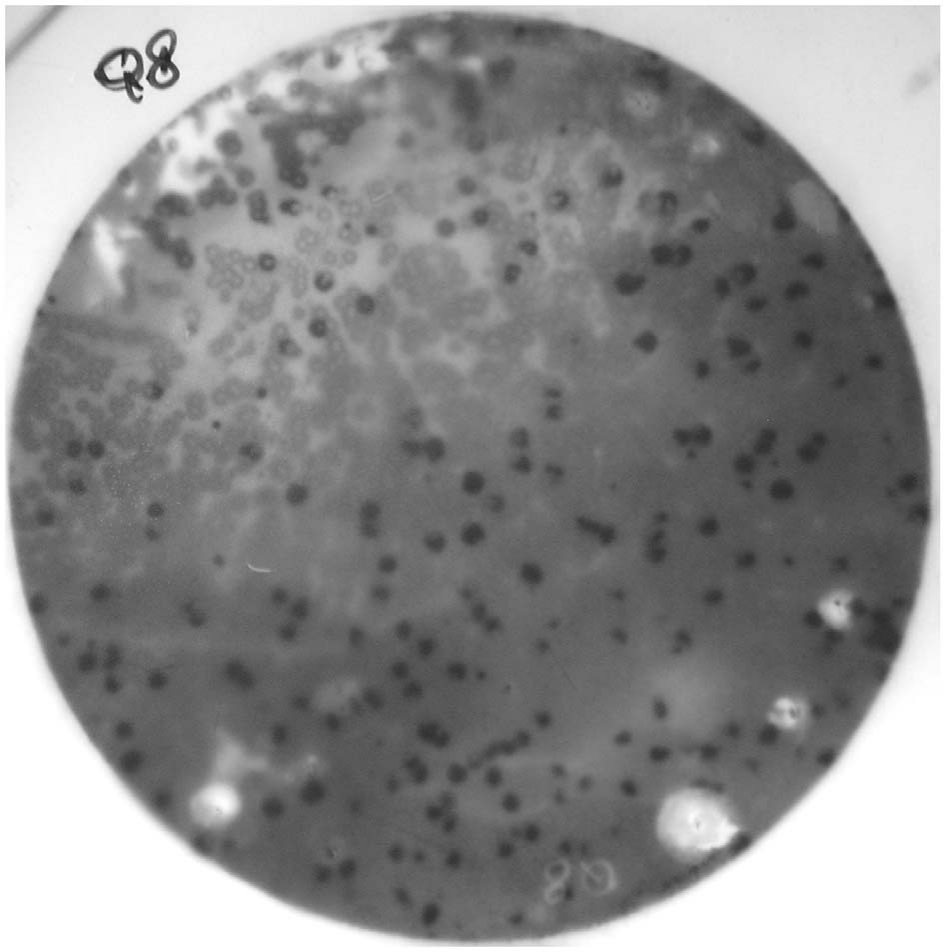
Second selection of positive clone. Many plaques from pickup of first positive signal have developed on another plate. Exogenous proteins produced by single plaques have been adsorbed to this filter membrane, and tested for the immunoreactivity with iMN patient serum. The difference between positive and negative plaques is apparent.

Clinical data at the time of biopsy included serum creatinine, glomerular filtration rate measured according to the MDRD formula [18], proteinuria (g/24 h), systolic and diastolic blood pressure.

Fifteen nephrotic patients affected by glomerulonephrites other than iMN or secondary MN were recruited (4 with diabetic nephropathy, 4 with focal glomerulosclerosis, 4 with type I membranoproliferative glomerulonephritis, 3 with minimal change disease). Histological diagnosis and collection of clinical data were performed following the same procedures as for the iMN patients. Fifteen normal subjects recruited among blood donors and matched to the iMN patients for age and sex were also recruited.

The samples were assigned numbers to render them anonymous. The institutional review board of our University Hospital approved the study and written informed consent was obtained from all of the subjects.

### Kidney Biopsy

Normal renal tissue was obtained by a cadaveric kidney donor.

Biopsies from patients recruited for the study were clinically indicated in order to assist with specific diagnosis. Biopsy was performed with a standard technique (see Methods S1).

**Figure 2:**
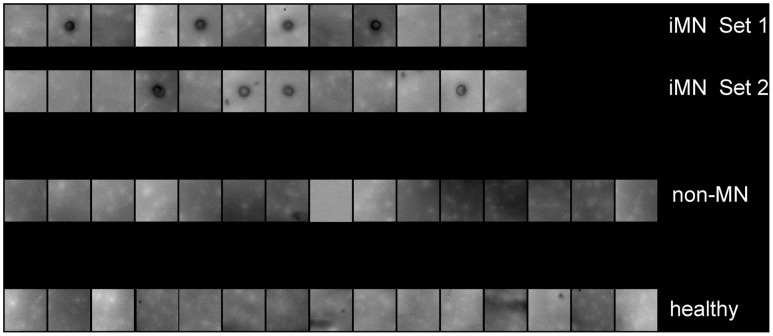
Recombinant spot assay of each serum. Positively reacting sera are those showing the dark spot in the middle of the membrane, where the recombinant SC65 protein was spotted.

### Immunofluorescence

OCT (Tissue Tek, Miles, Elkhart, IN) was the embedding medium for biopsy sample freezing in liquid nitrogen. Samples were subsequently cut into 3 µm sections by a cryostat (Leica 1720, Leica Mycrosystems, Heerbrog, Germany) and placed on glass slides (SuperFrost, Gerhard Menzel, Brunswick, Germany) for indirect immunostaining. Cryosections were fixed in modified Carnoy solution for 10 minutes at 4°C and subsequently washed in PBS. Non-specific binding was blocked by incubation in BSA 3% w/v in PBS for 20 minutes at room temperature. Sections were then incubated for 1 hour at 37°C with primary mouse anti-No55 antibody (BD Transduction Laboratories, BD Biosciences, San Jose CA, USA) and mouse anti-human IgG_4_ (Invitrogen Corporation, Carlsbad CA, USA) or mouse anti-Synaptopodin (Abnova Corporation, Taipei City, Taiwan) diluted 1∶10 in blocking solution. Alexa Fluor® 488 goat anti-mouse IgG_2a_ (1∶1000) and Alexa Fluor® 568 goat anti-mouse IgG_1_ (1∶1000) (Invitrogen) were used as secondary antibodies. Negative controls were processed in parallel using an equivalent concentration of a normal mouse antiserum as primary antibody.

### Preparation of Patient Sera

A 10 ml blood sample was collected from every subject early in the morning prior to biopsy and before the patient was indicated for immunosuppressive therapy. Within a few minutes from collection, serum was isolated by centrifugation (1000×g, 7 min) and stored immediately at –75°C. Prior to use, the serum samples were pooled in four groups: (i) from 12 iMN patients (discovery group), (ii) from other 12 iMN patients (confirmation group), (iii) from 15 non-MN renal patients (assorted as described), and (iv) from 15 healthy individuals. Preabsorption membranes were obtained by plating approximately 5,000 plaque forming units (pfu) of lambda phage carrying GAPDH cDNA and *E. coli* XL1-Blue MRF’ strain in NZY top agar and laying nitrocellulose membranes (Purabind 045 by Whatman, Dassel, Germany) previously soaked in 10 mM IPTG and air-dried.

Each serum pool was diluted 1∶100 in blocking solution (1× PBS, 3% BSA) and incubated with preabsorption membranes for five times.

**Figure 3:**
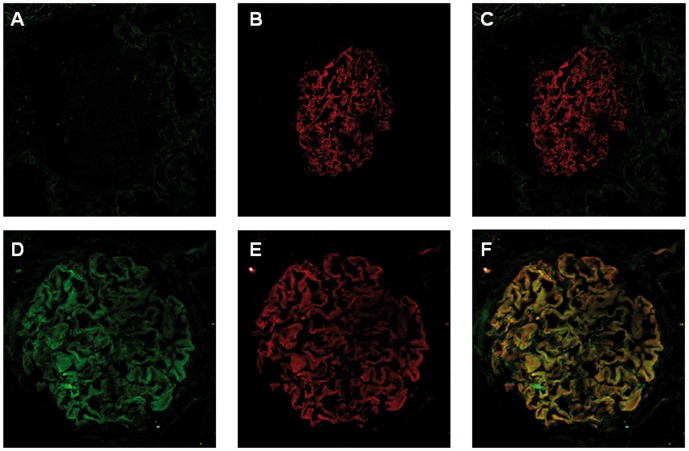
SC65 and Synaptopodin staining of normal and iMN renal tissue. The immunofluorescence image shows normal (A, B, C) and iMN kidneys (D, E, F) stained with monoclonal antibodies against SC65 (A, D) and synaptopodin (B, E). Pictures C and F result from the merge of A+B and D+E respectively. Costaining with synaptopodin show that SC65 and synaptopodin are co-expressed in the cytoplasm of podocyte cells.

**Figure 4:**
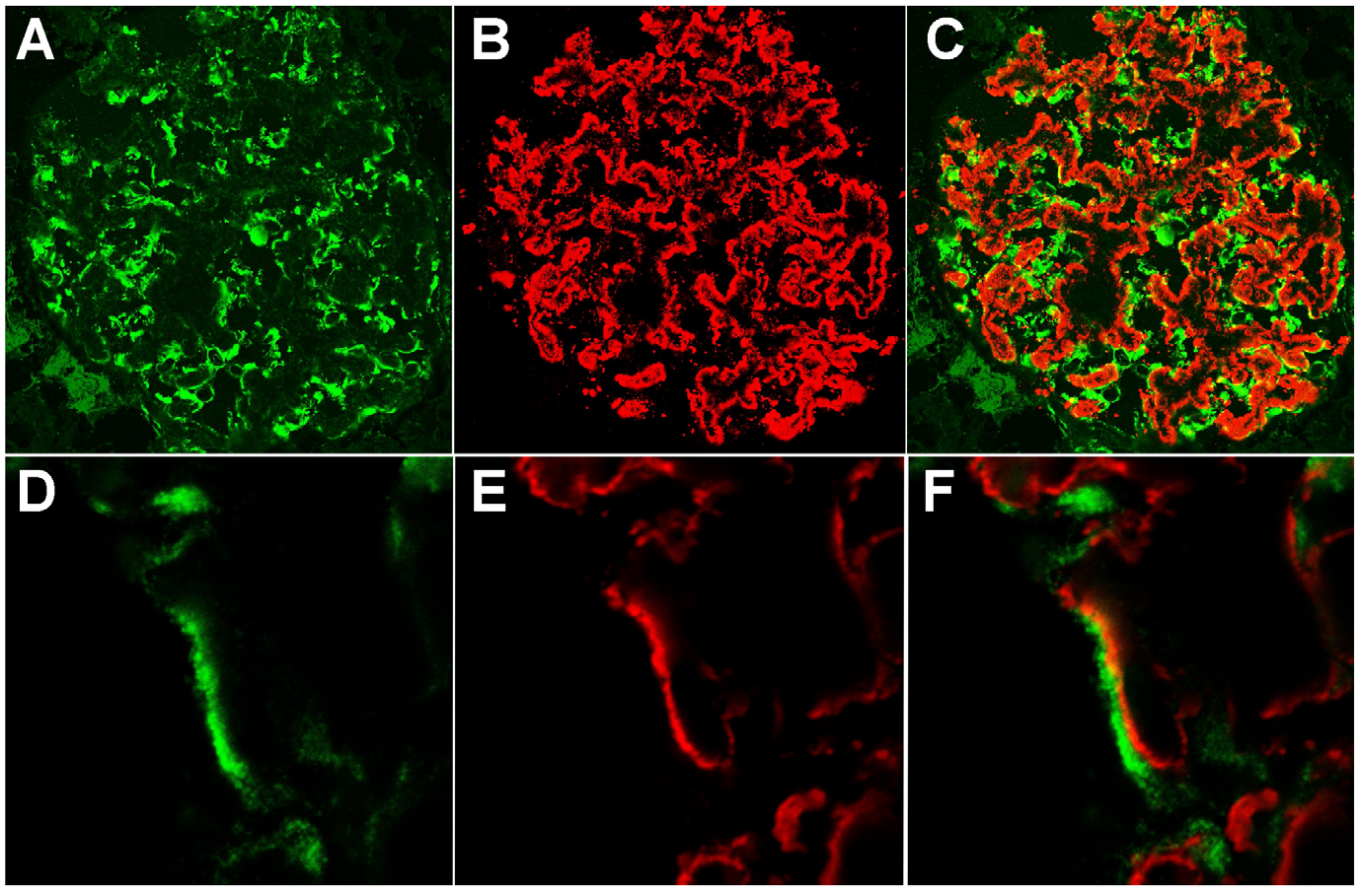
SC65 and IgG_4_ staining of iMN renal tissue. The immunofluorescence image shows an iMN kidney glomerulus (and a particular of it) stained with monoclonal antibodies against SC65 (A, D) and IgG_4_ (B, E). Pictures C and F result from the merge of A+B and D+E respectively. The costaining of SC65 and IgG_4_ deposits in iMN specimens shows a polarized expression of IgG_4_ in the subepithelial district adjacent to a cytoplasmic expression of SC65.

### Immunoscreening of the cDNA Expression Library

A commercially available 8 pooled kidney cDNA expression library (Stratagene, Cambridge, UK) was immune-screened with sera from the discovery phase group, according to the manufacturer’s protocol.

Positive plaques were re-screened with the same pool of sera to obtain the clonality. Afterwards, phages were recovered as pBluescript plasmids. See Methods S1 for further information.

**Figure 5:**
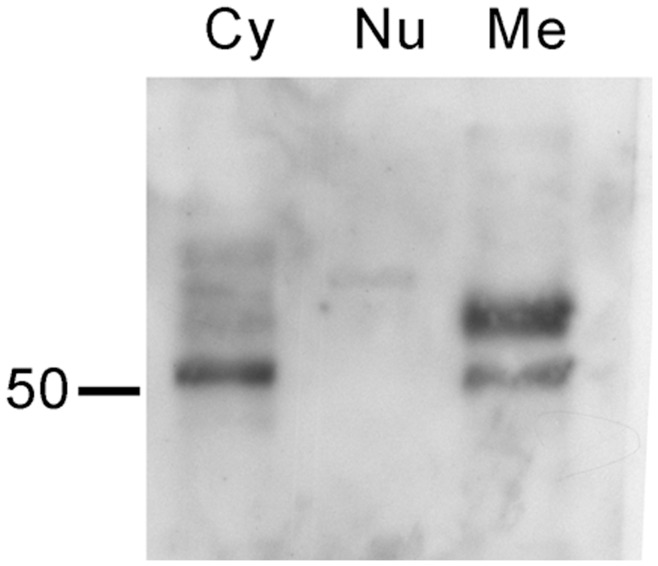
Western blotting of SC65 in subcellular fractions of cultured differentiated podocytes. **Cy**, cytosolic fraction. **Me**, membrane fraction. **Nu**, nuclear fraction. A band corresponding to SC65 is apparent in the cytosolic and membrane fractions.

### Serological Spot Assay

Screening of positive clones was carried out by a serological spot assay, as previously described [19]. In brief, 40 µl of exponentially growing *E. coli* XL1-Blue MRF’ were incubated with 40 µl of monoclonal phagemide containing 5,000 pfu per microliter; 0.7 µl of this mixture was spotted on a layer of NZY top agar (0.7% agarose) containing 2.5 mM isopropyl β-D-thiogalactoside laying on a NZY agar Petri plate. Once the mixture was set and the liquid absorbed by agar, a nitrocellulose membrane was laid on the plate and incubated at 37°C overnight. Therefore membranes were washed with TBST, blocked with 1× PBS 3% BSA and incubated overnight with blocking solution containing serum 1∶100, previously depleted of lambda phage and *E. coli* XL1-Blue MRF’. On control membranes, a mouse anti-human GAPDH monoclonal antibody (Santa Cruz Biotechnologies, Santa Cruz, CA) diluted 1∶4000 was used. Membranes were washed again with TBST and then incubated with sheep anti-human horseradish peroxidase-conjugated IgG antibody (or sheep anti-mouse horseradish peroxidase-conjugated IgG antibody in controls) (GE Healthcare, Little Chalfont, England) diluted 1∶4000 in 1× PBS for 1 h. A further cycle of TBST washing was applied. Antigen-antibody complexes were detected by ECL Western Blotting Detection System (GE Healthcare) and autoradiography film.

### Plasmid Excision and Isolation

Clonal plasmids were obtained from phagemids according to the manufacturer’s protocol. See Methods S1 for details.

Plasmids were then isolated following small-scale preparation protocol.

### Molecular Analyses

Plamids obtained as described were digested with *Bda*I and *Pvu*I restriction endonucleases and their inserts amplified for sequencing. See Methods S1 for further details.

### Sequencing and Identification

Sequencing was performed using the BigDye Terminator v1.1 Cycle Sequencing Kit (Applied Biosystems), and clone inserts were sequenced with an automated sequencer (ABI 3100). Sequence alignment was carried out by searching nucleotide databases with BLAST.

### Recombinant Spot Assay

Single sera were tested on spots of commercial recombinant protein SC65 (Abnova, Taipei City, Taiwan). Square 2-cm nitrocellulose membranes were prepared by spotting 0.5 µm of recombinant protein SC65 in the centre and blocked with 3% (w/v) BSA in 1× PBS for 1 h. Membranes were incubated overnight in PBS-diluted serum; they were thus washed three times in TBST and incubated with sheep anti-human IgG HRP-conjugated antibody 1∶4000 in PBS for 1 h. A further cycle of TBST washing was applied. Finally, immunocomplexes were detected by ECL Western Blotting Detection System and autoradiography film.

### Cell Culture

A human conditionally immortalized podocyte cell line [20] was cultured and proteins from subcellular fractions were obtained as previously described [7].

### Polyacrylamide Gel Electrophoresis and Western Blotting

Fifteen µg lanes of subcellular fractions were loaded on a standard 7.5% polyacrylamide gel in order to perform a gel electrophoresis. Migrated proteins were thus electroblotted onto a nitrocellulose membrane to undergo Western blotting analysis using a primary mouse anti-No55 antibody (BD Transduction Laboratories, BD Biosciences, San Jose CA, USA) diluted 1∶1000 in blocking solution, and a sheep anti-mouse horseradish peroxidase-conjugated IgG antibody as secondary antibody. The ECL Western Blotting Detection System was used to obtain images on the autoradiography film.

## Results

### Autoreactive Phagemide Plaque were Identified By Pooled Sera of Patients

Twenty-four nephrotic patients affected by iMN, 12 patients for the discovery phase and 12 for the confirmation phase were recruited for this work (see the Methods section for details on patient recruitment). The clinical characteristics of these patients are described in Table 1.

In order to identify possible autoreactive immune responses, pooled sera from 12 recruited iMN patients (discovery group) were used to probe a kidney cDNA expression library derived from normal kidney tissue whole mRNA. Sera were obtained at the time point of kidney biopsies, and iMN diagnosis was confirmed by immunofluorescence and histological procedures. Approximately 2×10^5^ recombinant clones of the library were screened by the pooled serum.

Six positive clones were detected. Immunoreactive phage plaques were then removed from the corresponding agar plates, and eluted phages were re-plated at lower plaque density and re-screened as above with patients sera to isolate specific, purified phage (Figure 1).

Putatively positive phage plaques were tested through a Serological Spot Assay to confirm the specificity of this immune reactivity. Pooled sera from 12 iMN patients (confirmation group), 15 control subjects and 15 patients affected by other glomerulonephrites than MN were used. A strong positive signal for iMN patients was found only from one out of six plaques (arbitrarily coded *Q8*).

### Q8 Identification by Restriction Fragment Analysis and Sequencing

After obtaining the pBluescript double stranded phagemid from the Q8 plaque, a restriction analysis using *Bda*I and *Pvu*I endonucleases was performed in order to excide the insert cDNA and estimate its size. Vector double digestion with *Bda*I and *Pvu*I endonucleases led to three fixed-length fragments (325, 1045 and 1158 base pairs) and one fragment whose length is given by cDNA insert plus 397 base pairs from flanking regions. The output of digestion was examined by agarose gel electrophoresis, allowing to infer that the putative length of cDNA fragment is about 2,600 base pairs.

The sequence analysis of the cDNA insert was performed by searching matches in the GenBank database through the BLAST algorithm. The search output pointed to a full identity with coding sequence of Synaptonemal Complex protein SC65 (accession number NM_006455), also known as Nucleolar Autoantigen (No55). The cDNA length in Genbank entry is 2619 bp, and the result is consistent with restriction analysis output. The identification was confirmed by the assessment of phagemid plaques expressing SC65 with monoclonal anti-No55 antibody.

### Recombinant Spot Assay

Each serum was evaluated with respect to its immunoreactivity to a spot of pure recombinant protein SC65 through an immunological analysis of recombinant protein spots. The sera of 24 iMN patients (control and validation sets), 15 non-MN patients and 15 healthy subjects were tested. Positive reactivity of 8 out of 24 patients suffering from iMN was found. None of the 15 non-MN patients and 15 healthy subjects showed reactivity against pure recombinant protein SC65 (Figure 2).

Considering that the clinical features iMN patients with a positive test showed lower albumin levels and a higher creatinine level, neither of these features reached statistical significance.

### Immunofluorescence

An indirect immunofluorescence staining of renal specimens using the monoclonal antibody against SC65 was carried out. Two commercial monoclonal antibodies raised against SC65 were tested: one of them resulted ineffective for any technique (data not shown). Thus the focus was particularly placed on the second antibody (see Methods), whose activity is certified for western blotting but not for immunofluorescence techniques. In consideration of these limitations, a significant SC65 signal was found in the glomeruli of patients affected by iMN; this pattern was substantially absent in the glomeruli of control tissue. Costaining with synaptopodin showed that SC65 is expressed in the cytoplasm of podocyte cells (Figure 3). The costaining of SC65 and IgG_4_ deposits in iMN specimens was also further investigated showing no clear match, but rather a polarized expression of IgG_4_ in the subepithelial district adjacent to a cytoplasmic expression of SC65 (Figure 4).

### Western Blotting

SDS-PAGE of cultured podocyte subcellular fractions was performed to find the possible localization of SC65 in the cell (Figure 5). Standard electrophoresis did reveal several bands, particularly in the cytoplasmic fraction, and in the membrane fraction as a double band. This is probably due to a post-translational modification of the protein migrating to the membrane. No band is observable in the nuclear compartment.

## Discussion

Membranous nephropathy is characterized by an accumulation of immune deposits, mainly of the IgG_4_ immunoglobulin subclass, on the outer aspect of the glomerular basement membrane. The debate on therapeutic management of patients affected by membranous nephropathy has been a regular theme not yet thoroughly addressed. [21], [22], [23] Steroids, cytotoxic drugs and anti-CD20 agents showed a significant benefit in selected cohorts of patients, reinforcing the hypothesis of a humoral autoimmune nature of the disease. [5], [6], [7], [9], [10], [11], [14], [15], [17], [24].

Nevertheless, the need for using such an unspecific therapy clearly provides evidence of an unsatisfactory knowledge of the underlying pathogenetic mechanisms. A more specific concept-driven therapy is thus urgently requested and obviously this cannot leave out the knowledge of the nature of immune deposits. We have conducted a clone library screening in the attempt to discover putative antigens representing the target of this autoimmune process.

Our work has pointed out a new protein as a potential candidate antigen for the iMN, i.e. Human Synaptonemal Complex protein SC65, also known as Nucleolar protein No55. By screening a phage library expressing renal proteins, we found a strong and quite specific reactivity for this antigen in the serum of patients affected by iMN. On the other hand, we could not detect the previously described autoantigens of iMN, probably because of the intrinsic limitations of protein representation in phage libraries. [25].

SC65 is a protein first characterized as an autoantigen in one case of interstitial cystitis (IC) in a patient not suffering from kidney diseases, and it was described as localized in the particulate compartment of the interphase nucleolus, with a distribution distinct from that of the nucleolar protein B23. [26].

Even if SC65 identified in the interphase nucleolus suggests a role in mitotic processes, an alternative role of this protein in meiosis was also debated: our data do not confirm the nucleolar localization of SC65; rather, we detected it in the non-nuclear compartments through histological evaluation and western blot analysis. SC65 expression was described in several normal human tissues: the highest mRNA expression levels were detected in ovaries, pancreas and prostate, whereas lower levels were retrieved in other tissues. Human renal expression has been demonstrated by Northern Blot analysis with an intermediate intensity between the low expression of the liver and the strong expression of the prostate. [27] An involvement of this protein has also been shown in patients suffering from prostate cancer as a tumour-associated autoantigen. [27].

Both autoimmune (IC) and cancer (prostate cancer) conditions were systematically investigated and excluded in the cohort of iMN patients recruited for this work. To our knowledge, the protein isolated in this study did not correspond to any candidate iMN antigen previously described. It is not clear which mechanism could drive the delivery of an intracellular protein to the extracellular space for its deposition beneath the glomerular basement membrane. Nonetheless, we cannot rule out a role for intracellular proteins and cryptic epitopes if we consider the possibility of exposition of these antigens after podocyte injury by C5b-9 attack complex. [17] Indeed, our results of immunofluorescence basal patterns suggest a newfangled localization of SC65, opening a parallel debate on the physiological function of this protein in the cell.

Despite the strongly significant serologic results, histological conclusions are not definitive for the lacking of clear subepithelial localization of the antigen. We cannot exclude a technical limit of our immunofluorescence histological analysis because the reagent has not been certified for this purpose. Still, the number of patients showing reactivity to the antigen is relevant and furthermore our data suggest a clinical meaning for this association. Indeed positive patients showed lower albumin and higher creatinine levels. We can speculate that positive patients express a more clinically relevant and aggressive disease, and this hypothesis is under further investigation.

In conclusion, our report describes the finding of anti-SC65 antibodies in patients affected by iMN. The role of this antigen under the diagnostic and prognostic value would be ascertained by an extensive analysis of a large cohort of iMN patients that is already in progress.

## Supporting Information

Methods S1 (DOC)
