# Supplementary material for: Identification and Characterization of a New Autoimmune Protein in Membranous Nephropathy by Immunoscreening of a Renal cDNA Library
Source: PLoS One. 2012 Nov 8;7(11):e48845. doi: 10.1371/journal.pone.0048845 (PMC3493607; doi:10.1371/journal.pone.0048845)
Supplement: Methods S1 — (DOC) [file pone.0048845.s001.doc]

**Supplementary Material**

**Subjects and Methods**

*Patients and Control Subjects*

Twelve nephrotic patients affected by iMN for the discovery phase and twelve nephrotic patients affected by iMN for the confirmation phase were recruited. The diagnosis of iMN was made on the basis of biopsy examination using immunofluorescence techniques and optical microscopy according to parameters of the ‘*World Health Organization Centre for the Histological Classification of Renal Diseases, 1982*’. Patients with the following characteristics were included in this study: biopsy proven iMN, age older than 18 years, proteinuria > 2 g/day, creatinine < 1.8 mg/dL. Patients affected by connective tissue diseases, positive to ANA, ENA antiDNA, antiphospholipids antibodies, ANCA, diabetes mellitus, neoplasia, Hepatitis B or C virus infection, HIV or systemic diseases, patients younger than 18 years at the time of biopsy were excluded.

Clinical data at the time of biopsy included serum creatinine, glomerular filtration rate measured according to the MDRD formula^17^, proteinuria (g/24h), systolic and diastolic blood pressure.

Fifteen nephrotic patients affected by glomerulonephrites other than iMN or secondary MN were recruited (4 with diabetic nephropathy, 4 with focal glomerulosclerosis, 4 with type I membranoproliferative glomerulonephritis, 3 with minimal change disease). Histological diagnosis and collection of clinical data were performed following the same procedures of the iMN patients. Fifteen normal subjects recruited among blood donors and matched for age and sex to the iMN patients were also recruited.

The samples were assigned numbers to render them anonymous. The institutional review board of our University Hospital approved the study and written informed consent was obtained from all of the subjects.

*Kidney Biopsy*

A cadaver kidney explant that was not suitable for transplantation due to the incidental discovery of a neoplastic nodule of 5 cm at the lower pole upon macroscopic examination was used as normal renal tissue. The pathologic examination diagnosed a clear cell carcinoma (grade 2 according to Fuhrman classification; T1bN0M0 according to the International Union Against Cancer classification); the lower pole was excised and the remaining parenchyma was used for the following procedures. The remaining parenchyma did not reveal any evidence of clear cell carcinoma.

Biopsy samples were examined by light and immunofluorescence microscopy (BX41 Microscopy, Olympus). The histological features were recorded on the base of sections stained by haematoxylin-eosin, periodic acid/Schiff, Masson’s trichrome and Silver.

Biopsies from patients recruited for the study were clinically indicated in order to assist with specific diagnosis. Biopsy was performed at the lower renal pole of the left kidney by an ultrasound assisted technique (ATL APOGEE 800 PLUS Ultrasound machine) and local anaesthesia. A manual Tru-Cut biopsy kit with a 16 gauge, 150 mm length needle (Urocore; HS Hospitalservice, Aprilia, Italy) was used. Two specimens for every patient were collected: one for light microscopy, and the other one for immunofluorescence. All subjects provided informed consent prior to biopsy. The patients were on bed rest for 24 hours after biopsy and subsequently underwent an ultrasound examination to exclude renal bleeding.

*Immunofluorescence*

OCT (Tissue Tek, Miles, Elkhart, IN) was the embedding medium for biopsy sample freezing in liquid nitrogen. Samples were afterwards cut into 3 µm sections by a cryostat (Leica 1720, Leica Mycrosystems, Heerbrog, Germany) and placed on glass slides (SuperFrost, Gerhard Menzel, Brunswick, Germany) for indirect immunostaining. Cryosections were fixed in modified Carnoy solution for 10 minutes at 4°C and subsequently washed in PBS. Nonspecific binding was blocked by incubation in BSA 3% w/v in PBS for 20 minutes at room temperature. Sections were then incubated for 1 hour at 37°C with primary mouse anti-No55 antibody (BD Transduction Laboratories, BD Biosciences, San Jose CA, USA) diluted 1:10 in blocking solution. FITC-conjugated goat IgG anti-mouse IgG_2a_ (1:1000) was used as secondary antibody. Negative controls were processed in parallel using an equivalent concentration of a normal mouse antiserum as primary antibody.

*Preparation of patient sera*

A 10 ml blood sample was collected from every subject early in the morning before the biopsy and before the patient was indicated for immunosuppressive therapy. Within a few minutes from collection, serum was isolated by centrifugation (1000×g, 7min) and stored immediately at –75°C. Prior to use, the serum samples were pooled in four groups: (i) from 13 iMN patients (discovery group), (ii) from other 13 iMN patients (confirmation group), (iii) from 15 non-MN renal patients (assorted as described), and (iv) from 15 healthy individuals. Preabsorption membranes were obtained by plating approximately 5,000 plaque forming units (pfu) of lambda phage carrying GAPDH cDNA and *E. coli* XL1-Blue MRF’ strain in NZY top agar and laying nitrocellulose membranes (Purabind 045 by Whatman, Dassel, Germany) previously soaked in 10 mM IPTG and air-dried.

Each serum pool was diluted 1:100 in blocking solution (1× PBS, 3% BSA) and incubated with preabsorption membranes for five times.

*Immunoscreening of the cDNA expression library*

*E. coli* XL1blueMRF' cells were infected with a commercially available 8 pooled kidney cDNA expression library (Stratagene, Cambridge, UK) and plated in NZY top agar to an approximate density of 10,000 pfu/plate on 20 NZY agar plates. Therefore 200,000 pfu were screened.

After 3 h 30’ of incubation at 42°C, the fusion protein expression was induced by lying on the plate nitrocellulose membranes which were previously soaked in 10 mM IPTG. The plates with the filters were stored overnight at 4°C subsequently to a second incubation for 3 h 30’ at 37°C. Membranes were removed, washed three times for 15 min in 1× TBST (0.1% Tween) and blocked with 3% (w/v) BSA in 1× PBS for 1 h. Membranes were thus incubated overnight in diluted first iMN patients pooled serum prepared as described, then washed three times for 10 min in 1× TBST and incubated with sheep anti-human IgG antibody (GE Healthcare, Little Chalfont, England) conjugated to horseradish peroxidase diluted 1:4000 in 1× PBS for 1 h. A further cycle of TBST washing was applied. Antigen-antibody complexes were detected by ECL Western Blotting Detection System (GE Healthcare) and autoradiography film.

Positive plaques were re-screened with the same pool of sera to obtain the clonality. Phages were recovered as pBluescript plasmids by double-stranded rescue using the ExAssist helper phage (Stratagene) according to the manufacturer's instructions and used to transform SOLR cells.

*Serological Spot Assay*

Screening of positive clones was carried out by a serological spot assay, as previously described(14). In brief, 40 µl of exponentially growing *E. coli* XL1-Blue MRF’ were incubated with 40 µl of monoclonal phagemide containing 5,000 pfu per microliter; 0.7 µl of this mixture was spotted on a layer of NZY top agar (0.7% agarose) containing 2.5 mM isopropyl β-D-thiogalactoside laying on a NZY agar petri plate. Once the mixture was set and the liquid absorbed by agar, a nitrocellulose membrane was laid on the plate and incubated at 37°C overnight. Therefore membranes were washed with TBST, blocked with 1× PBS 3% BSA and incubated overnight with blocking solution containing serum 1:100, previously depleted of lambda phage and *E. coli* XL1-Blue MRF’. On control membranes, a mouse anti-human GAPDH monoclonal antibody (Santa Cruz Biotechnologies, Santa Cruz, CA) diluted 1:4000 was used. Membranes were washed again with TBST and then incubated with sheep anti-human IgG antibody conjugated to horseradish peroxidase (or sheep anti-mouse horseradish peroxidase-conjugated IgG antibody in controls) (GE Healthcare, Little Chalfont, England) diluted 1:4000 in 1× PBS for 1 h. A further cycle of TBST washing was applied. Antigen-antibody complexes were detected by ECL Western Blotting Detection System (GE Healthcare) and autoradiography film.

*Plasmid excision and isolation*

Clonal plasmids were obtained from phagemids according to manufacturer’s protocol. In brief, phage suspension was joined to *E. coli* XL1-Blue MRF’ suspension and ExAssist helper phage suspension in LB broth and incubated at 37°C for 3 hours. Bacteria were inactivated at 70°C for 20 minutes and debris was discarded by centrifugation. Filamentous phages present in suspension were thus incubated with *E. coli* SOLR strain and plated on LB-ampicillin agar plates. Overnight incubation at 37°C resulted in colonies of bacteria containing pBluescript plasmids carrying the cDNA sequence.

Plasmids were then isolated following small-scale preparation protocol.

*Molecular analyses*

8 µg of pBluescript obtained as described were incubated overnight with 1 U of *Bda*I restriction endonuclease at 30°C, and for 2 hours with 5 U of *Pvu*I at 37°C.

Digestion products were later run on 1.5% agarose gel.

PCR was carried out with the GeneAmp XL PCR Kit (Applied Biosystems, Foster City, CA) in a thermal cycler (Applied Biosystems GeneAmp PCR System 9700) for 10 cycles. Initial hot start denaturation was at 94°C for 5 min; each cycle consisted of incubation for 15 s at 94°C, annealing for 30 s at 58°C and extension for 5 min at 72°C. This protocol was repeated for 20 more cycles adding 15 s per cycle to the extension step. Final extension was carried out for 10 min at 72°C.

Primer sequence was studied on pBluescript multiple cloning site flanking regions: forward primer sequence was 5’-GTTTTCCCAGTCACGACGTT-3’; reverse primer sequence was 5’-ACCATGATTACGCCAAGCTC-3’.

PCR product was purified with ExoSAP-IT (USB Corporation, Cleveland, OH) according to manufacturer’s instructions.

*Sequencing and Identification*

Sequencing was performed using the BigDye Terminator v1.1 Cycle Sequencing Kit (Applied Biosystems). The primers used were the same as the PCR ones.

Purification was carried out by blending 10 µl of PCR product with 2 µl of 3M Na-acetate and 50 µl of absolute ethanol. The mix was then centrifuged at 13,000 g for 30 min at 4°C. The pellet was washed in 70% ethanol and again centrifuged for 5 min. The pellet was then air dried.

Clone inserts were sequenced with an automated sequencer (ABI 3100). Sequence alignment was carried out by searching nucleotide databases with BLAST.

*Recombinant Spot Assay*

Single sera were tested on spots of commercial recombinant protein SC65 (Abnova, Taipei City, Taiwan). Square 2 cm nitrocellulose membranes were prepared by spotting 0.5 µm of recombinant protein SC65 in the centre and blocked with 3% (w/v) BSA in 1× PBS for 1 h. Membranes were incubated overnight in PBS-diluted serum; thus they were washed three times in TBST and incubated with sheep anti-human IgG HRP-conjugated antibody 1:4000 in PBS for 1 h. A further cycle of TBST washing was applied. Immunocomplexes were then detected by ECL Western Blotting Detection System and autoradiography film.

*Cell Culture*

A human conditionally immortalized podocyte cell line^18^ was cultured and proteins from subcellular fractions were obtained as previously described^7^.

*Polyacrylamide gel electrophoresis and Western blotting*

Fifteen µg lanes of subcellular fractions were loaded on a standard 7.5% polyacrylamide gel in order to perform a gel electrophoresis. Migrated proteins were thus electroblotted onto a nitrocellulose membrane to undergo Western blotting analysis using a primary mouse anti-No55 antibody (BD Transduction Laboratories, BD Biosciences, San Jose CA, USA) diluted 1:1000 in blocking solution and a sheep anti-mouse horseradish peroxidise-conjugated IgG antibody as secondary antibody. The ECL Western Blotting Detection System was used to obtain images on autoradiography film.
